# Best Clinical Practice in Botulinum Toxin Treatment for Children with Cerebral Palsy

**DOI:** 10.3390/toxins7051629

**Published:** 2015-05-11

**Authors:** Walter Strobl, Tim Theologis, Reinald Brunner, Serdar Kocer, Elke Viehweger, Ignacio Pascual-Pascual, Richard Placzek

**Affiliations:** 1 Department of Paediatric- and Neuro-Orthopaedics, Orthopaedic Hospital Rummelsberg, 90592 Schwarzenbruck, Germany and MOTIO, 1080 Vienna, Austria; E-Mail: walter.strobl@sana.de; 2 Department of Orthopaedics, Rheumatology and Musculoskeletal Sciences, Nuffield Orthopaedic Centre, Windmill Road Headington, Oxford OX3 7LD, Oxfordshire, UK; E-Mail: timtheologis@googlemail.com; 3 Department of Paediatric- and Neuro-Orthopaedics, University Children’s Hospital Basel (UKBB), Spitalstrasse 33, 4056 Basel, Switzerland; E-Mail: reinald.brunner@ukbb.ch; 4 Centre de Réadaptation de COUBERT (Ugecam) 77170, France and ROMATEM, Etiler Istanbul 34337, Turkey; E-Mail: skocer1@gmail.com; 5 Department of Paediatrics, Hospital Infantil La Paz, Universidad Autonóma de Madrid, Madrid, Spain; E-Mail: sipascual@telefonica.net; 6 Department of Paediatrics, Hospital Infantil La Paz, Universidad Autonóma de Madrid, Madrid, Spain; E-Mail: sipascual@telefonica.net; 7 Orthopedic Department, University Hospital Bonn, Sigmund-Freud-Str. 25, 53127 Bonn, Germany

**Keywords:** botulinum toxin, BoNT-A, Cerebral palsy, child development, spasticity, treatment recommendation

## Abstract

Botulinum toxin A (BoNT-A) is considered a safe and effective therapy for children with cerebral palsy (CP), especially in the hands of experienced injectors and for the majority of children. Recently, some risks have been noted for children with Gross Motor Classification Scale (GMFCS) of IV and the risks are substantial for level V. Recommendations for treatment with BoNT-A have been published since 1993, with continuous optimisation and development of new treatment concepts. This leads to modifications in the clinical decision making process, indications, injection techniques, assessments, and evaluations. This article summarises the state of the art of BoNT-A treatment in children with CP, based mainly on the literature and expert opinions by an international paediatric orthopaedic user group. BoNT-A is an important part of multimodal management, to support motor development and improve function when the targeted management of spasticity in specific muscle groups is clinically indicated. Individualised assessment and treatment are essential, and should be part of an integrated approach chosen to support the achievement of motor milestones. To this end, goals should be set for both the long term and for each injection cycle. The correct choice of target muscles is also important; not all spastic muscles need to be injected. A more focused approach needs to be established to improve function and motor development, and to prevent adverse compensations and contractures. Furthermore, the timeline of BoNT-A treatment extends from infancy to adulthood, and treatment should take into account the change in indications with age.

## 1. Introduction

Cerebral palsy (CP) is the most frequent cause of spasticity in children [1]. CP is the result of cerebral lesions occurring in the pre-, peri- and post-natal period. Recent magnetic resonance imaging (MRI) studies suggest that common abnormalities include periventricular white matter lesions, focal ischaemic/haemorrhagic lesions, diffuse encephalopathy, basal ganglia damage and brain malformations [2,3]. CP may be regarded as a static brain lesion causing a permanent motor impairment with evolving musculoskeletal manifestations. 

Over the past two decades botulinum toxin type A (BoNT-A) has been established as an important treatment modality for spastic movement disorders in children with CP. In most countries worldwide, it is licenced for children older than two years, but that licencing and labelling varies dramatically from one country to another. Due to this fact, the majority of BoNT-A use is “off label”. Today, BoNT-A is only one part of the multi-disciplinary model of managing such patients. Other treatments include functional therapies (physiotherapy, occupational therapy, speech therapy, constraint-induced movement therapy, robotic-assisted therapy, *etc*.); orthoses, casting and splinting; pharmacotherapies; intrathecal baclofen; selective dorsal rhizotomy (SDR); and single-event multi-level orthopaedic surgery, including the minimal invasive and other surgical reconstructive techniques [4].

**Figure 1:**
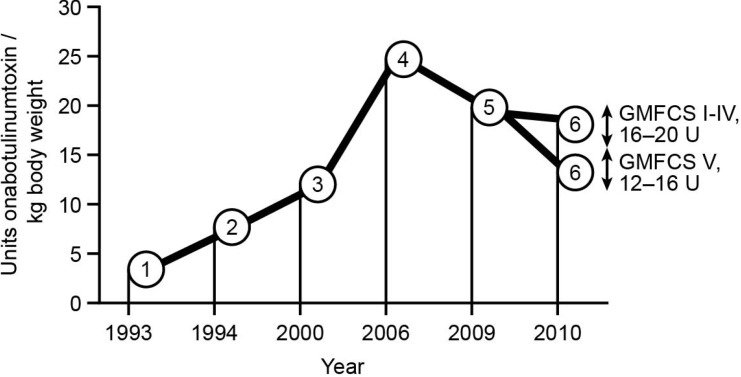
Reported/recommended total dose of botulinum toxin A (onabotulinumtoxinA) for the treatment of infantile cerebral palsy [4,5,7,8,9,10].GMFCS, Gross Motor Function Classification System. 1. 2–4 units [7]; 2. 5–10 units [8]; 3. 12 units [9]; 4. 25 units [10]; 5. 20 units [4]; 6. 12–16 units [5] for GMFCS V; 16–20 units* for GMFCS I–IV; unitsonabotulinumtoxinA/kg body weight.

There is no uniform BoNT-A treatment strategy in CP, and the doses used have varied significantly over the years. The increase in the recommended total doses of BoNT-A over recent years, measured in units/kg body weight, is shown in Figure 1. These total doses are not evidence-based, but are based on “expert opinion” and mostly small clinical trials of investigators. A useful safety work by the manufacturers, for example in primates, is missing until today. Randomised, double-blind, placebo-controlled, dose-ranging studies are rare with BoNT-A. The recommended dosage in Europe was recently reduced due to a better understanding of the relationship between dosage, severe side effects and the type of anaesthesia used [5,6].

Within the current clinical management of CP in children, the use of BoNT-A is recommended to improve function and to support motor development [11]. Recent recommendations take into account the severity of the treated children, according to the Gross Motor Function Classification System (GMFCS) [5]. A number of national guidelines on BoNT-A use in CP [12,13,14], and updated European and international consensus statements [4,5] have been published. We are not in full agreement with these guidelines and are concerned over the ever-increasing dosage of BonT-A. Therefore, we are suggesting a new approach based on reviewing the literature and collecting the opinions of experts within an international board. We have summarised this as best clinical practice.

## 2. Methods

The authors reached a consensus about BoNT-A treatment of children with CP following an initial meeting in 2011, and subsequently through cross-review of the present article, ending with a final meeting in 2014. A literature review was conducted by searching MEDLINE (PubMed) using the terms “recommendations”, “guidelines” and “established treatment concepts” and the key words “botulinum toxin” and “cerebral palsy” with date limiters of January 1990 to May 2014. The searches were conducted in May 2014.

## 3. Indications for BoNT-A Use 

The management needs of children with different types of CP vary, and the indications for BoNT-A in CP are summarised in Table 1. BoNT-A treatment is used when the targeted management of spasticity in specific muscle groups is clinically indicated. Defining the indications for such management decisions is a complex process which requires the rigorous assessment of the individual child by a multi-disciplinary team. History and clinical examination are of paramount importance. The specific problems of the individual child, any pain, the level of motor function and the potential for further development should be taken into account. The views of the parents/carers and the input of other health professionals involved in the management of the child should also be taken into account. Finally, it is important to set specific goals for the treatment before initiating BoNT-A therapy.

In the German guidelines for spasticity management, published by the German Society for Neurology, treatment of focal spasticity is recommended due to the high level of evidence. In several European countries, BoNT-A is licensed for spasticity as a symptom independent of underlying disease [15]. For localized/segmental spasticity in the upper and lower extremities of children with CP that warrants treatment, BoNT-A should be offered as an effective and generally safe treatment (Level A) [16].

**Table 1:** The most common indications for botulinum toxin A treatment in unilateral, bilateral ambulant and bilateral non-ambulant children with cerebral palsy.

Localization	Unilateral CP	Bilateral ambulant CP	Bilateral non-ambulant CP
Upper limb	Improved function and aesthetics/appearance	N/A	Pain management
			Easier caring and positioning
			Functional and/or cosmetic improvement of hand position
Lower limb	Improved gait	Improved gait	Pain management
			Easier caring and positioning
			Improvement of weight bearing
			Prevention of hip dislocation
Spine	N/A	N/A	Postural management
			Care
			Pain management

CP, cerebral palsy; N/A, not applicable.

Generic indications for BoNT-A use should be seen as guidelines only and are not necessarily to be followed strictly. Since the severity and distribution of the neurological impairments vary significantly among children, the targets of BoNT-A treatment vary accordingly. An important challenge in assessing a child and in planning BoNT-A treatment is the identification of the specific problematic muscle activity to be targeted. This relies not only on rigorous evaluation of the child but also on relevant clinical experience and expertise. 

In younger children, the motor development is still evolving, and therefore BoNT-A treatment usually targets spastic muscles which are impeding development, in order to facilitate therapy goals and maximise the child’s potential. The timing of such intervention is also dictated by the severity of the neurological involvement; more severely involved children are likely to require earlier treatment. In older children, where motor development is largely completed and motor patterns are established, BoNT-A can be used again to target specific muscles in order to maximise function and reduce pain and spasticity. Specific functional goals should be set and the appropriate muscle selection should be made following careful assessment. If the functional aim is to improve gait, for example, a detailed analysis of the child’s walking pattern would be necessary in order to identify the target muscles. Following successful attainment of functional goals through BoNT-A treatment, repeated injections over longer periods of time are used to maintain the benefit and to prevent or delay the development of fixed contractures (e.g., by facilitating physiotherapy and the fitting of orthoses) in the targeted muscles but evidence is still lacking [17].

In the context of specific goal attainment, BoNT-A is used under a wide variety of indications, including the facilitation of physiotherapy stretching, the fitting of orthoses and casts and the management of spasm-related pain, including post-operative pain. BoNT-A is used to treat severely involved non-ambulant children in order to improve posture and care. Functional gains can sometimes be considered (e.g., improved positioning of the upper limb for better control of powered wheelchairs or communication devices). There is disagreement amongst physicians regarding the use of BoNT-A in the prevention or delay of hip dislocation.

Classification according to GMFCS is widely used and helpful in defining indications for BoNT-A treatment. However, both the severity of neurological involvement and the anatomical distribution of the neurological involvement are important considerations when planning BoNT-A therapy. Combining those two parameters, three separate groups of children with cerebral palsy can be considered: unilateral, bilateral ambulant and bilateral non-ambulant. Table 1 shows the most usual indications for BoNT-A treatment in each of the three groups, but this list of indications is not exhaustive.

An additional factor to be considered is the “trigger muscle phenomenon” which suggests that injecting the appropriate trigger muscles has a beneficial influence on spasticity of non-injected muscles. This is based on neuromuscular interactions between ipsi- and contralateral proximal and distal muscles [18]. 

## 4. What Is an Effective and Successful Treatment?

The assessment of treatment outcome depends entirely on the goals defined before treatment. When treatment is used to improve a specific function, for example, this function should be assessed and measured before and after treatment to provide an objective and measurable evaluation of the effectiveness of treatment. Therefore, successful treatment relies on realistic and achievable goal setting, and this should be planned at the appropriate time for the individual child [19,20].

Tone reduction in a specific muscle group, achieved through the administration of BoNT, does not, in itself, define effective treatment unless it is combined with an optimal clinical result. In equinus gait, for example, BoNT-A treatment of the gastrocnemius may reduce tone and spasticity, but may not correct the equinus gait if there are other contributing impairments (e.g., hamstrings spasticity and increased knee flexion). In this context, goal attainment (correction of equinus gait) should be considered as failed and the treatment should be considered ineffective [20]. In controlled studies of pes equinus show high dosages no advantage to medium doses and injection intervals of four months seemed to be more effective than annual injections [21,22]. However, assessment of the effectiveness of treatment in reducing tone in the targeted muscle(s) should form part of the overall assessment of the outcome. In this example, it is important to know if treatment failed because the muscle did not respond (e.g., because of fixed contracture or dystonia) or because of an erroneous assessment of the aetiology of equinus gait. In order to test this idea, an accurate clinical examination including distinguishing spasticity from contracture is absolutely necessary.

## 5. The Importance of Goal Setting, Assessment, and Evaluation

Any treatment intervention in children with complex neurological disability should be driven by specific aims and goals. BoNT-A treatment is no exception to this principle. The goals of treatment should be agreed between the child and family and the health professionals involved in his/her care. Multi-disciplinary assessment should precede these decisions. The information and views provided by the patient’s family and carers should be taken into consideration. Physicians and surgeons assess children for relatively short periods of time, usually in the hospital environment. Therefore, therapists involved in the regular treatment of children over time may have a more complete view of the individual child’s progress and potential [23,24]. Medical professionals of different specialities may bring different aspects of expertise in the assessment of the child and their opinions are all of significant value [25]. It cannot be stressed enough that it is the agreed aim and goal that define the nature of treatment.

The timing of intervention is also of paramount importance. This should take into account the progress of the child, his/her age and the severity of the neurological picture. It is again the multi-disciplinary team who should recommend the most suitable time for intervention. Patient characteristics, including patient personality, family support and the availability of therapy and other health professionals’ support should be considered. 

Treatment goals can be broadly grouped as follows:

- Improvement of function, including gait.
- Improvement of posture.
- Pain management.
- Facilitation of care.

However, goals should always be patient-specific and realistic. It is often useful to define short-term (for each injection cycle) and long-term goals, as well as primary and secondary goals, and these must be reassessed regularly. Repeated assessment after each injection cycle would aid the decision of whether further treatment with BoNT-A should be pursued. The long-term approach should aim to obtain or improve the patient’s gross motor function.

## 6. The Importance of an Integrated Approach

An essential component of any BoNT-A treatment concept is the multimodal treatment approach, which may include physiotherapy or orthoses, among the range of other treatments. When relevant, patient-centred goals have been set and BoNT-A treatment is potentially indicated based on severity and age, the place of BoNT-A within the integrated treatment should be assessed, since the timing of other treatments will influence the timing of BoNT-A treatment.

The integration of BoNT-A treatment with other therapies can be illustrated with some of the examples below. Many treatment combinations may be indicated:

- Physiotherapy (especially stretching and strengthening) and occupational therapy (in the upper limb) combined with BoNT-A therapy is more beneficial than occupational and physiotherapy alone [22], and is recommended in patients receiving BoNT-A therapy [5,26,27].
- There are limited data on the benefits of combining BoNT-A therapy with orthoses [28], and one study has suggested that orthoses may not be as beneficial as casting when used as part of multimodal treatment involving BoNT-A therapy [29]. Reduced muscle tone may be best treated with stabilising orthoses.
- There are also conflicting data on the benefits of combined casting and BoNT-A therapy *versus* either treatment alone [30,31,32], but recent reports suggest the combination is beneficial [33,34,35].
- Orthopaedic surgery has an important role in the treatment of the musculoskeletal deformities and contractures present in the child with CP. The widely accepted principle is the single event multi-level surgery. One of the roles of BoNT-A therapy is to avoid multiple operations in order not to weaken muscles excessively and to protect children from multiple admissions in hospital. The challenge is to time the surgery correctly for the individual child in order to avoid going back. Perioperative BoNT-A injections may help to reduce spasticity-induced post-operative pain and to ease the rehabilitation process. BoNT-A injection may also help to confirm surgical indications: if the patient deteriorates functionally after injecting the target muscles, any planned surgery to these muscles should be approached with caution [36].
- SDR may reduce spasticity in selected individuals. There is a role for BoNT-A therapy in the long-term follow-up of SDR in many children [37].
- In cases of severe generalised spasticity, the combination of oral tone-reducing medications or intrathecal Baclofen treatment and BoNT-A may have a therapeutic effect.

## 7. Development of Different Treatment Concepts 

Although the therapeutic potential of BoNT-A was described as early as 1973 [38,39], its use in the treatment of focal spasticity in children with CP was first reported in the early 1990s [7,8]. In these early reports, treatment was based on a single-level approach such as for the treatment of dynamic equinus. Low-dose injections have shown clinical effects [7]. Increased experience in the BoNT-A treatment led to the use of increasingly higher dosages within the single-level treatment context [8].

They were soon replaced by multi-level injections (the injection of several muscles and muscle groups at each injection session, for example the gastrocnemius, medial knee flexors, adductors and psoas), as these could improve lower limb joint alignment [9]. Some form of multi-level treatment is required in most children with CP, since ≥80% of them have multi-level problems [40]. Medium-dose, multi-level regimens were shown to carry benefits for the natural history of equinus foot deformity, ankle function, walking, grip strength, and overall functional ability [41,42,43,44,45]. Furthermore, these studies suggested that higher doses were more effective than the lower doses used in the initial studies [9,46]. A key part of this medium-dose, multi-level concept is that early initiation of BoNT-A therapy may be preferable [47,48,49].

A high-dose, multi-level concept has been widely used in the last decade, despite there being no generally agreed procedure. This concept is based on the injection of each target muscle at each level in cases with more severe spasticity [10,50]. As BoTN-A is distributed across several muscle groups, systemic side effects are rare and the safety profile was reported to be good [51,52]. However, there have been isolated reports of severe side effects with high-dose BoNT-A therapy [53,54,55]. 

Recommendations from 2010 have suggested a reduction in total doses. [5] Earlier initiation of BoNT-A treatment has also been assessed and proposed (Figure 1) [56]. Differentiation of dosage according to GMFCS levels has been established in recent years. Severity of disease and possible effect of sedation might have an influence on the development of side effects [57]. Individualisation of dose is crucial to success. However, recent recommendations underestimate the effectiveness of lower dose concepts; they can also be effective and probably more safe [58,59]. 

## 8. An Integrated Treatment Approach: The Key-Muscle Concept

In order to provide the best possible support for motor development at each individual motor milestone and using moderate dose recommendations, the key-muscle concept was developed. This concept is a refinement of the high-dose multi-level concept and consists of an advanced multimodal therapeutic approach [60,61]. The key-muscle concept aims to better address the particular characteristics and complexity of CP. If children with CP achieve a higher level of motor development through BoNT treatment, they benefit from this development for longer than they would solely from the pharmacological effect of BoNT.

BoNT-A therapy should have the following characteristics: long-term applicability; sustainability; and individual and flexible planning. A basic requirement for long-term treatment is the avoidance of secondary non-response and the formation of antibodies to BoNT. Thus, within the context of the key-muscle concept, multi-level injections are performed that strictly comply with well-proven dosage recommendations:

- AbobotulinumtoxinA: ≤20 units/kg body weight for the first injection and subsequent injections of ≤30 units/kg body weight with a maximum total dose of 1000 units abobotulinumtoxinA, following the European Marketing Authorisation.
- OnabotulinumtoxinA: ≤12 units/kg body weight for the first injection and subsequent injections ≤15 units/kg body weight with a maximum total dose of 300 units onabotulinumtoxinA, following conservative recommendations [46,62].
- IncobotulinumtoxinA: ≤12 units/kg body weight for the first injection and subsequent injections of ≤15 units/kg body weight with a maximum total dose of 300 units (assuming dose equivalence of 1:1 between onabotulinumtoxinA and incobotulinumtoxinA [63].

The key-muscle concept is characterised by: the treatment goal being the next stage of physiological motor development; selection of the key muscles; early commencement of treatment; and long-term treatment.

### 8.1. Treatment Goal

**Figure 2:**
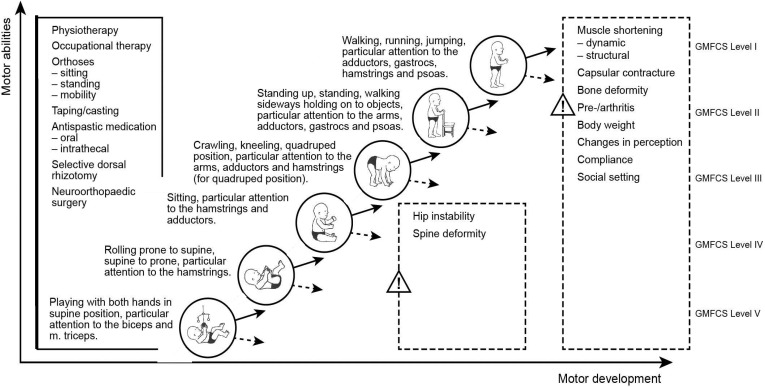
Physiological development/motor milestones with available therapy options listed in the left box. To every milestone, the affected muscles (key muscles for Botulinum toxin injection) are displayed. In case of stagnation (dashed arrows), secondary alterations and deformities are shown in the right box. GMFCS level descriptors can be viewed separately as supplementary material.

Reaching the next motor milestone—with the prospective goal of standing and weight bearing and achieving the best possible locomotion—is the primary aim. If a child gains the ability to walk, the goal is to maintain, improve and optimise mobility. In the case of stagnation on a lower motor level, the goal is to maintain, improve and optimise motor function on this level. The motor milestones have been defined according to Petö [64], the GMFM [65], and World Health Organization (WHO) classification [66] as shown in Figure 2.

### 8.2. Selection of Key Muscles

Key muscles are those muscles that, due to spasticity, prevent attainment of the next motor milestone. Additionally, muscles at immediate risk for contracture or even muscles with early contracture are injected. Spastic muscles do not have to be injected if the pathologic tone does not impair function if there is no acute risk of developing contractures, or if the tone allows for compensatory mechanisms.

### 8.3. Early Commencement of Treatment

The rationale for starting treatment as early as possible is based on the finding that in the first year of motor development, voluntary movements are organised around behavioural objectives. The underlying idea of advanced neurorehabilitation is based on neuroplasticity and it is probable that a younger brain has a higher potential for motor learning [67,68,69]. Children try out a combination of manoeuvres to achieve a goal, and thus, they learn new and more rapid movements, and improve their coordination. To attain stable function, repetitive performance is necessary. In children with CP, there may be an incorrect combination of movements. As a consequence, multiple repetitions lead to non-physiological learning, which results in non-physiological motor development or neglect of the affected extremity. In addition, non-physiological movements may lead to persisting effects in a developing brain [67]. During physiological development of the brain, there is a progressive reduction of the cortical area activating motor function and hence an increase in the selective and specific control of movement [68]. If non-physiological movement patterns are not learned in the first place, ‘dead ends’ of motor development may be avoided. Normally, all five motor milestones are reached in the first two years of life. The first years of life are also important in spasticity-related hip lateralisation and dislocation [70]. A recent review states an advantage of BoNT-A treatment in children younger than two years, for reducing spasticity, avoiding contractures and postponing surgery. However, clear evidence regarding the improvement in general motor development cannot be derived. There is need for further randomized controlled trials as well as the development of assessment tools that are reliable and valid for infants of this age [71].

The safety profile of the recommended doses of BoNT-A is the same for children under two years as for older children [72]. Early BoNT-A treatment of muscles prone to shortening helps to prevent fixed contractures so that the number and extent of later surgical interventions can be reduced [49]. Sufficient physiotherapeutic support is essential for the success of early treatment according to the key-muscle concept [73].

### 8.4. Long-Term Treatment

A basic requirement for the long-term treatment option is to avoid secondary non-response and formation of antibodies. A specific timescale for integrated BoNT-A treatment is given in Figure 2. 

## 9. Clinical Examination, Assessment, Evaluation and Documentation

Monitoring of the patient, and assessment of treatment effectiveness and achievement of goals, involve the use of appropriate tools. The choice of the appropriate outcome measure depends on the goal that has been set. If the goal is to improve gait, for example, objective gait assessment should be undertaken before and after the intervention. The same assessment tool should be used before and after treatment to allow meaningful assessment of outcome and objective evaluation. Some suggested outcome tools are discussed below, but these are examples only and a variety of alternative assessment tools are available. It is important, however, to select outcome measurement tools that have been appropriately validated for use in the specific field.

Routine clinical examination prior to BoNT-A injection includes:

- Active and passive range of motion.
- Specified muscle testing e.g., Thomas-test, popliteal angle, Silverskiöld, Duncan-Ely.
- Muscle strength according to Janda, MRC, Oxford-scale.
- Observational gait analysis supported by video documentation [74].
- Goal attainment scale (GAS) [75,76].
- Modified Ashworth Scale (MAS) is simple and reproducible in the assessment of muscle spasticity, but is probably of limited validity [77,78].
- Modified Tardieu Rating Scale is more reliable, and is focused on most clinically relevant parts of the Tardieu Scale [23,79,80].
- Gross Motor Function Measure (GMFM) is a validated tool for the measurement of motor function in children with CP [58,65]. It may, however, not be sensitive enough to detect the minimal changes that occur following relatively minor interventions.
- Upper limb assessment tools e.g., SHUEE and AHA scores or PEDI [81,82,83].
- Activities Scale for Kids (ASK), only applicable to children aged 5–15 years [84].
- Three-dimensional instrumented gait analysis has been invaluable in order to document function before and after BoNT-A injection, which may be used as an objective parameter to assess gait (in cases where gait improvement is the aim). It is also extremely useful in planning surgical management and as an outcome measure in clinical studies [16,85,86].

However, application of these assessment tools in everyday clinical practice may be limited by cost and availability. 

Quality of life (QoL) impairments, and potential improvements with treatment, must be measured in children with CP. However, although many quality of life assessment tools have been used in CP and many of these are validated in several languages, a reliable measure cannot be recommended [59,87]. The most informative method to include in outcome studies appears to be self-evaluation of quality of life by children with CP, using a questionnaire based on patients’ and families’ opinions, in association with a participation questionnaire. 

Recent studies for children’s QoL detect a difference in the outcome of children’s and their parents’ surveys, when grading the QoL with and without CP [88,89].

To reduce the risk of errors, a checklist should be used with the consent of the patient. This checklist may include the patient’s identity, indication, contra-indications (e.g., anticoagulant medication, pregnancy, motor neuron disease), date of the last injection (including bladder injection), and which muscles, side and dose to inject.

## 10. Critical Considerations

Spasticity is associated with weakness, which affects stability and motor function. The main decision for the use of BoNT-A is to distinguish whether high muscle tone impedes or improves function. A further difficulty is to test whether a specific muscle is slightly spastic or just being used. Stiffness may be caused by avoiding involuntary muscle movements owing to weakened muscles, or by simultaneous activity of a group of muscles with high muscle tone. Neither dynamic EMG during gait nor gait analysis was capable of providing a clear parameter related to spasticity [90,91]. These study results indicate that currently the decision on whether to treat with BoNT-A is a clinical one, and relies less on the assessment of individual muscles and more on the general functional impairment. The clinical assessment, using tools such as the Ashworth Scale, Modified Tardieu Scale, or Range of Motion, is less important in the use of BoNT than the general functional and developmental assessment and outcome. For this reason, it is essential to define the goal of treatment and to assess the result with an appropriate test. This test should reflect the rehabilitative goal and not only the effect on the injected local muscles only. An objective assessment is beneficial for patients, parents and caregivers, since BoNT-A injections invoke a desire to see a positive physiological effect.

There is a general agreement that recovery is always complete. However, up to now there have been no reports detailing the long-term effects of BoNT-A has on treated muscles. Although the application of BoNT-A seems to be safe and effective, long-term studies are necessary and patient-based assessments are lacking.

## 11. Application of BoNT

Today, three different BoNT-A preparations are available. Specific dosages in different preparations are not interchangeable. There are several studies evaluating the dosages and comparability of different preparations, but without any consensus [92,93,94]. BoNT-A passes easily through muscle fascia even at subclinical doses, but the presence of fascia reduces distribution of BoNT-A by 23% [95]. Distribution is necessary to reach enough motor endplates. It can be controlled by two factors: concentration of medication, and injected volume. It is important in suspected non-response to differentiate dynamic contracture from structural contracture. Only dynamic contracture can be treated with BoNT. With increasing age, structural contractures are increasing. Application of recent treatment concepts with moderate dosages avoids antibody formation.

## 12. Optimising Injection

Efficacy and safety of BoNT-A depend on dose and application and should be adjusted based on indication and clinical assessment. In animal studies, described long-term effects on the muscular skeletal system, in the sense of degradation, underline the need for moderate dosages in an elaborated treatment strategy such as the key-muscle concept [96,97]. Dose optimisation and best practice in application are crucial. The injection technique has to be reliable, comfortable and easy to use in routine practice. Prerequisites are adequate instruments like needles and guiding techniques, experience and optimal clinical setting.

Unsatisfactory outcomes may be due to incorrect indications, wrong choice and missed target muscles as well as a lack of complementary treatment (e.g., rehabilitation or splinting).

### 12.1. Techniques to Guide Injection

Several techniques are available to guide injection, including palpation, ultrasound, electromyography (EMG) or electrical stimulation. By using palpation and anatomical landmarks, target muscles can be identified. The technique described by Cosgrove, to observe needle move while passive motion of body segment, may be easily used for localisation of the needle in the target muscle [98]. Correct needle placement guided by palpation has shown no difference in clinical outcome compared with other techniques [99,100].

Ultrasound is a real-time, dynamic imaging method that does not involve ionising radiation, and has excellent spatial resolution. It is, therefore, well suited to interventional guidance. Being a low-cost examination, ultrasound guidance provides rapid and reliable identification of target muscles, even deep-seated ones such as the iliopsoas [101,102]. The neighbouring structures are visible, reducing the risk of iatrogenic misplacement of the injections, and this method provides information about the depth, echo-structure and volume of the muscle. There is good acceptance of the technique in young children and it may also be useful in sedated patients [103]. A high frequency linear probe (≥7.5 mHz) and common needles are used; however, identification of visualized structures requires training and experience.

For EMG-guided injection, a standard or portable EMG machine may be used, and a Teflon^®^-coated EMG needle is recommended. Target muscles can be identified by recording the motor unit potentials. Identification by EMG is well adapted for focal dystonia. However, spastic muscle groups in CP do not allow the differentiation of single target muscles.

Electrical stimulation uses an EMG machine or portable stimulator together with a Teflon^®^-coated EMG needle, and elicits contraction of the target muscle. 

### 12.2. Where to Inject

In theory, BoNT-A is more efficient if injected near the motor endplate areas. In striated muscle fibres, the motor endplates are located at the midpoint of the fibre which can be found in mammalian muscles using a canine model. Childers *et al*. showed that better localisation of motor endplates using EMG guidance led to a greater drop in mean muscle force generated at two and five weeks post-injection. Most of the lower limb muscles have a complex fibre structure [104]. As a result, several sites of injection at the mid-muscle belly are needed for optimal treatment.

## 13. Conclusions

BoNT-A therapy is considered an effective and clearly safe treatment for children with CP in the hands of experienced injectors and in the management of the majority of children with GMFCS I–III. Higher risks have been noted recently for children with GMFCS IV and more substantial for GMFCS V. It is an important part of multimodal management: to support motor development and improve function when the targeted management of spasticity in specific muscle groups is clinically indicated. Individualised assessment and treatment are essential prior to and following injection, and should be part of an integrated approach that will support the achievement of motor milestones. To this end, goals should be set for each injection cycle and for the long-term. The correct choice of target muscles is also important: not all muscles suffering spasticity need to be injected; a more focused approach is needed to improve function and motor development, and to prevent adverse compensations and contractures. Injection technique is important for the success and safety of BoNT-A treatment. Furthermore, the timeline of BoNT-A treatment extends from infancy to adulthood, and treatment should take into account the change in indications with age.
